# Accurate prediction of dynamic viscosity of polyalpha-olefin boron nitride nanofluids using machine learning

**DOI:** 10.1016/j.heliyon.2023.e16716

**Published:** 2023-05-26

**Authors:** Yazeed AbuShanab, Wahib A. Al-Ammari, Samer Gowid, Ahmad K. Sleiti

**Affiliations:** Department of Mechanical & Industrial Engineering, College of Engineering, Qatar University, Qatar

**Keywords:** Nano-fluids, Dynamic viscosity, Adaptive neuro-fuzzy inference, Artificial neural network, System, Prediction, Correlation

## Abstract

This study focuses on predicting the dynamic viscosity of nanofluids, specifically Polyalpha-Olefin-hexagonal boron nitride (PAO-hBN) using machine learning models. The primary goal of this research is to assess and contrast the effectiveness of three distinct machine learning models: Support Vector Regression (SVR), Artificial Neural Networks (ANN), and Adaptive Neuro-Fuzzy Inference System (ANFIS). The main objective is the identification of a model that demonstrates the highest level of accuracy in predicting a nanofluid’s viscosity namely, PAO-hBN nanofluids. The models were trained and validated using 540 experimental data points, where the mean square error (MSE) and the coefficient of determination R^2^ were utilized for performance evaluation. The results demonstrated that all three models could predict the viscosity of PAO-hBN nanofluids accurately, but the ANFIS and ANN models outperformed the SVR model. The ANFIS and ANN models had similar performance, but the ANN model was preferred due to its faster training and computation time. The optimized ANN model had an R^2^ of 0.99994, which indicates a high level of accuracy in predicting the viscosity of PAO-hBN nanofluids. The elimination of the shear rate parameter from the input layer improved the accuracy of the ANN model to an absolute relative error of less than 1.89% over the full temperature range (−19.7 °C–70 °C) compared to 11% in the traditional correlation-based model. These results suggest that the use of machine learning models can significantly improve the accuracy of predicting the viscosity of PAO-hBN nanofluids. Overall, this study demonstrated that the use of machine learning models, specifically ANN, can be effective in predicting PAO-hBN nanofluids’ dynamic viscosity. The findings provide a new perspective on how to predict the thermodynamic properties of nanofluids with high accuracy, which could have important applications in various industries.

## 1 Introduction

Nanofluids are relatively new materials used to boost the thermodynamic performance of many engineering systems including solar energy systems, machines that run on electricity, and manufacturing processes [1]. Improving the performance of energy systems can have several benefits, including reducing adverse environmental effects, decreasing energy consumption, and lowering costs. These factors are crucial for achieving sustainable energy systems. Sustainability approaches have been used to assess the economic and environmental performance of nanofluids in various energy systems and to determine their potential advantages. Nanofluids show great promise in heat transfer applications, particularly in response to the increasing demand for cooling applications [2].

Many studies have been conducted to examine the effect of nano-fluids on various applications. For example, the effect of water/CuO nano-fluids has been examined on the flow and heat transfer measurements of a turbulent flow, where it is shown that the nanofluids improved the overall heat transfer of the base fluid [3]. Similarly, a study was conducted to measure the hydrothermal performance of a porous sinusoidal double-layered heat sink using a silver water-based nanofluid. The study examined multiple cases, and in all instances, the hydrothermal performance was improved [4]. Another example, an Al_2_O_3_ water-based nanofluid was used as a working fluid of a flat solar collector which enhanced its efficiency by 22% compared to the conventional collector [5]. In other reports, Abdallah et al. tested a nanofluid (multi-walled carbon nanotubes) in a photovoltaic application which led to an overall efficiency of 61% [6]. The fundamental benefit of using nano-fluids is that system performance can be improved without major changes like modifying a system's conventional dimensions or adjusting technical requirements [7]. However, their use is restricted due to the difficulty of predicting their thermodynamic properties.

Nanofluids play a key role in the sustainability of several engineering systems and research areas including solar energy systems [8], heat exchanger design [9], and machining cutting fluids [10]. For instance, a study conducted by Liu et al. [11] showed that the use of nanofluids enhanced the efficiency of the solar collector by 25%. Another example, using ZnO nano-fluid, the heat transfer coefficient of a shell and tube heat exchanger is enhanced by 40% [12]. The performance of nanofluids in various energy applications has been extensively reviewed in the literature, with a primary focus on assessing the sustainability of different types of nanofluids [[13], [14], [15], [16], [17]]. These reviews have different conclusions depending on the field that nanofluids and nanotechnology are used. For instance, nanofluids have proved to be a favorable solution for sustainability and performance in desalination applications. On the other hand, further experimental analyses are needed to further validate the sustainability of using nanofluids in the field of concentrated solar power.

Nanofluids have many thermodynamic properties that must be determined precisely to harvest their benefits in practical applications. Thus, different correlations were introduced to predict their thermal properties using experimental methods with an emphasis on thermal conductivity and viscosity. For example, A recent research investigation examined a ternary hybrid nanofluid’s thermal conductivity. The study observed the relationship between conductivity, temperature, and volume fraction, providing valuable insights into their interdependence [18]. In a related experimental study, a hybrid non-Newtonian nanofluid composed of MWCNTs-ZnO dispersed in a mixture of water and ethylene glycol was investigated to establish a correlation for viscosity. The aim was to investigate the link between various components and the viscosity behavior of the nanofluid. Different correlations were found depending on the temperature range (25 °C–50 °C) and volume fraction range (0.075%–1.2%) [19].

These correlations cover several types of nanofluids, including both oil-based and water-based variants. However, these correlations suffer from poor accuracy and large deviations between them [20]. On the other hand, artificial neural network (ANN) models (such as those based on Feedforward, Multilayer perceptron, Radial, Recurrent, and Modular approaches [21]) have proved to be useful tools in such complex applications. These techniques heavily depend on data and past patterns to predict and forecast the present and future features of interest. The next subsections present a brief overview of ANN-based studies that were performed for the prediction of the nanofluid thermal properties, with a particular focus on dynamic viscosity.

The implementation of artificial intelligence (AI) techniques is a relatively new topic in the research field, these techniques include ANN [22], CNN [23], SVM [24], and ANFIS [25]. They are used in various research fields such as soil mechanics and properties [26], hospitalization [27], strength and properties of materials [28], risk assessment and prediction [29], and energy consumption in buildings [30]. In particular, it is reported that from all research done on nanofluids in 2019, only 3% are related to AI and neural networks [31]. Recently, a valuable review by Wang and Chen [32] presented how the machine learning approach was used for the prediction of thermodynamic properties of nanofluids. It comprehensively analyses literature published in the last decade (up to 2021). Thus, herein, a brief review is presented for the latest studies in 2022. It was noticed that most of these studies investigate the ANN approach and compare the results with correlation-based results as in Refs. [[33], [34], [35], [36]]. Other studies have investigated the ANN alongside other machine learning approaches including Boosted Regression Tree (BRT), and Support Vector Machine (SVM) [37], or other statistical tools such as response surface methodology (RSM) [38]. For example, He et al. [39] studied the efficacy of an ANN model in the prediction of conductivity of Zinc Oxide–Silver (50%–50%)/Water hybrid Newtonian nanofluid. Results show a low mean absolute error of 0.0095.

As shown in Table 1, the thermal/dynamic viscosity of the nanofluids is predicted based on several parameters including the size [36,40,41], volume fraction (VF) [40], and shape [42] of the nanoparticles as well as the temperature and base fluid type. In addition, some studies use the nanofluid density as input parameter to predict the viscosity as in Refs. [35,41,43]. In general, it can be noted that the nanofluid viscosity is predicted using at least two inputs (temperature and VF). In addition, the use of these ANN models may be limited to a certain temperature and VF ranges. Furthermore, most of the ANN models provide accurate predictions with R^2^ higher than 0.99. Also, other data-driven approaches are used and provide similar accuracy such as the Physics Guided Deep Neural Network (PGDNN) approach that is implemented by Bhaumik et al. [41] with R^2^ of 0.9961.

**Table 1:** Summary of recent studies (2022) that apply the ANN approach for prediction of nano-fluids^a^ thermal/dynamic viscosity.

Refs.	Nano-fluid(s)	Methods	Input variables	R^2^ of ANN	Remarks
[40]	Al_2_O_3-_WB	ANN	VF, Size, Sonication time, Temp.	0.9999	For volume fractions between 0.01% and 0.1%.
[44]	MWCNT-Al_2_O_3_-OB	ANN	Temp., VF, SR	0.9000	The sensitivity analysis evaluated the impact of temperature, solid volume fraction (SVF), and shear rate (SR) on viscosity of nanofluid (μ_nf_)
[37]	EGB nano-fluids	BRT, SVM, ANN	Temp. VF	0.9971	The BRT showed slightly better results than ANN and SVM.
[33]	MWCNT-MgO-OB	ANN, Correlation	Temp., VF, SR	0.9990	ANN showed better performance compared to correlation (R^2^_correlation_ = 0.9321)
[45]	MWCNT-ZnO hybrid-OB	ANN	Temp., VF, SR	0.9900	Single configuration was studied
[46]	WO_3_-MWCNT-EGB	ANN	Temp., VF	0.9980	Different training algorithms are evaluated alongside “trainlm”
[34]	MWCNT-ZnO-OB	Correlation, ANN	Temp., VF, SR	0.9921	A single hidden layer with six neurons was studied only. The ANN showed better performance than the correlation
[35]	TiO_2_-Al_2_O_3_-EGB	Correlation, ANN	Temp., VF, Density	0.9982	Slight better performance for the ANN over correlation.
[36]	CuO-EGB	Correlation, ANN	Temp., VF, Size	0.9997	Viscosity measurements of volume concentrations of 1%–4% are conducted in the temperature range of 293 K–353 K
[38]	MWCNT-OB	ANN, RSM	Temp., VF, SR	NA	Four optimization case studies were conducted, focusing on objective functions derived from nanofluid (NF) applications in various industries
[41]	Al_2_O_3_, CuO, SiO_2_, TiO_2_	PGDNN	Density, Size, VF, Temp., μ_base-fluid_, μ_simulated_,	0.9961	PGDNN approach integrates data-driven models with physics-based theoretical models to leverage their complementary strengths and enhance the modeling of physical processes
[47]	Al_2_O_3_, MWCNT, GNP-WB	MLPNN	Temp., VF, Nanofluid	0.9998	For VFs of (0.1%, 0.25%, 0.5%, 0.75%, and 1%) and temperature range 30–80 °C
[48]	ZrSiO_4_	ANFIS	Temp., VF	1	C-clustering is used in obtaining membership function.
[49]	Fe_3_O_4_-coated MWCNT	ANFIS, GEP	Temp., VF	0.9702	Genetic algorithm was used and studied.

a WB = water based, OB = oil based, VF = volume fraction, SR = shear rate, EGB = Ethylene glycol based, MWCNT = multi-walled carbon nanotubes.

From Table 1, the viscosity investigations were focused on nanofluids containing conventional nanoparticles such as Al_2_O_3_, MWCNT, ZnO, CuO, SiO_2_, and TiO_2_. However, a new class of nano-fluids is made by the combination of Polyalpha-Olefin (PAO) oil with hexagonal Boron Nitride (hBN) [50]. These nano-fluids are used in cooling and lubrication applications. Boron nitride nanofluids that have water as their base fluid have been found to exhibit improved performances in heat transfer and cooling applications due to their favorable thermophysical properties [51]. This makes them a popular choice in the field. Additionally, PAO is widely used as a lubricant and cooling fluid in various industries [52]. Therefore, combining PAO with BN to create nanofluids has the potential to further enhance cooling and lubrication performance.

It is noted that the available correlations for the thermal properties of PAO-hBN nano-fluids are limited. In particular, their dynamic viscosity is investigated in only one study that is presented by Sleiti [50]. This study presents a correlation that estimates the viscosity in terms of the nano-fluid concentration and fluid temperature. However, at some volume fractions, its performance is not accurate as the error may exceed 7% relative to the experimental values. In general, the experimental measurements of these properties are difficult and face many limitations including cost, time, and effort [53]. Therefore, the main objectives of this study are:

• Developing a machine learning based model for the prediction of dynamic viscosity of oil-based PAO-hBN nano-fluids.
• Investigating the performance of three machine learning models, namely Support Vector Regression (SVR), Artificial Neural Networks (ANN), and Adaptive Neuro-Fuzzy Inference System (ANFIS), in forecasting the viscosity of PAO-hBN nanofluids.
• Comparing the performance of the machine learning-based model with the only available correlation that is presented by Sleiti [50].

The rest of the manuscript is organized as follows: Section 2 describes the principles of three machine learning-based methods that are used in this study. Then, the configurations of these methods are explained in Section 3. The performance of these methods with a comparison to the available correlation in Ref. [50] is discussed in Section 4. A summary of the main outcomes and conclusions of this research are presented in Section 5.

## 2 Machine learning-based prediction methods

This section introduces a brief description for the machine learning-based methods that are used in this study.

### 2.1 Support vector regression (SVR)

Support vector regression (SVR) is one of the subsets of support vector machine (SVM) that is used for non-linear regression problems [54]. The main idea of SVR is to map the behavior of the output as the input changes. The fundamental concept of SVR is to find the optimum fit line. In a complex multi-input model, the optimum line is represented as a hyperplane, where this hyperplane has the maximum number of points (true value) on it. However, SVR differs from other methods in how its training works, rather than typical error value reduction, it tries to fit this hyperplane within a minimum threshold value. This threshold is the margin (distance between the hyperplane and boundary lines). The fit is not just exclusive to a polynomial fit, as it becomes a very tough task to fit and map an accurate model with larger datasets. Linear and gaussian support vector is recommended for larger datasets due to its quickness and low training time. However, general support vector does not consider predictions which are highly accurate for training points where the error is higher and trains on those data subsets. In ideal cases SVR is expected to produce the output y=f(x), where the error (ε) is minimal. The SVR generates a function to predict the relationship between input and outputs regardless of the nature of the relationship. This function is shown in Eqn. (1) [55].

(1) $$f\left(x,w\right)=\sum_{i}^{l}wh_{i}\left(x\right)+b$$

where w is a weight coefficient, b is the bias, and $h_{i}\left(x\right)$ is the function generated by the model. Both w and b are provided through a minimizing a risk function, such as given in Eqn. (2) [55]. The error (ε) is responsible for generating the slack variables (ξ_i_ and ξ_i_∗) which represent the upper and lower bound of the error. These slack variables transform the model at hand to an optimization model where the objective function is shown in Eqn. (3) and the constraints are given in Eqns. (4), (5) [56].

(2) $$R\left(c\right)=C\frac{1}{l}\sum_{i=1}^{l}L_{c}\left(y_{i}f\left(x_{i},w\right)+\frac{1}{2}{\left\|w\right\|}^{2}\right)$$

(3) $$\frac{1}{2}{\left\|w\right\|}^{2}+C\sum_{i=1}^{n}\xi_{i}+\xi_{i}^{*}$$

(4) $$y_{i}-\left(wh_{i}\left(x_{i}\right)+b\right)\leq \xi_{i}+\xi_{i}^{*}$$

(5) $$x_{i}+b-y_{i}\leq \xi_{i}+\xi_{i}^{*}\;;\;where\;\xi_{i}\xi_{i}^{*}\geq 0$$

To account for the intricate nature of certain relationships and models, it is advisable to select specific kernel functions that align with the intended application. In this study non-linear kernel functions are applied and evaluated including polynomial and gaussian which are available in the MATLAB framework.

### 2.2 Artificial neural network (ANN)

ANNs are computational models with a mathematical basis that try to imitate the human neural system/unit and learns similar to the way the nervous system of a human [57]. ANNs are designed to process parameters simultaneously, enabling them to identify complex relationships and changes among input variables and data. Furthermore, even when data is not precise and some of it is difficult to understand ANN goes a step further by detecting nonlinear complex relationships accurately and reliably. The main general steps to employ and implement an ANN to any application are as follows: (a) data processing and cleaning (b) data division into training, validation, testing (optional) (c) data normalization (d) determining the ANN structure and configuration including mainly number of inputs, training algorithm, layers hidden, neurons per layer, learning rate, outputs. It should be noted that data normalization is needed when two or more input variables are on dramatically different scales.

The structure of ANN is built upon direct linkage between inputs, outputs, and no less than one hidden layer [58]. Each hidden layer normally consists of two or more artificial neurons (nodes). A numerical value called bias is given to each layer and a weight is given to each neuron. Different training algorithms, such as Levenberg-Marquardt (LM), resilient propagation (RP), and Bayesian regularization (BR), utilize a nonlinear transfer function to transmit values from one layer to the next within a neural network. In the ANN computations, the sum of the input with its weights and bias is related to the hidden neurons, this is then passed on to the activation function which passes the new value to each neuron. Every neuron’s output at any specified layer is given as shown in Eqn. (6) [59]:

(6) $$y_{ij}=f_{j}\left[\sum_{i=1}^{q}w_{ij}X_{i}+b_{j})\right]$$

where $y_{ij}$ is the output of the neuron *i* of the layer *j*, $f_{j}$ is the activation function of layer *j*, $w_{ij}$ is the weight of each variable, $X_{i}$ is the input vector, and $b_{j}$ is the bias of the layer *j*. The non-linear activation function ($f_{j}$) is expressed in different forms such as sigmoid (binary and bipolar), ReLU, identity function, hyperbolic tangent, and linear function. For a continuous output (such as the viscosity of nano-fluid), the sigmoid activation function (Eqn. (7)) is recommended for the hidden layers and the identity function is used (Eqn. (8)) for the output layer.

(7) $$f\left(x\right)=\frac{1}{\left(1+e^{-x}\right)}$$

(8) f(x)=x

Different statistical metrics are typically used for the evaluation of the ANN model performance including mean square error (MSE) and the coefficient of determination (R^2^), which are shown in Eqns. (9), (10):

(9) $$MSE=\frac{1}{N}\sum_{I=1}^{N}{\left(y_{i}-{\hat{y}}_{i}\right)}^{2}$$

(10) $$R^{2}=1-\frac{\frac{1}{N}\sum_{I=1}^{N}{\left(y_{i}-{\hat{y}}_{i}\right)}^{2}}{\frac{1}{N}\sum_{I=1}^{N}{\left(y_{i}-{\bar{y}}_{i}\right)}^{2}}$$

where $y_{i}$, ${\hat{y}}_{i}$, ${\bar{y}}_{i}$, and *N* are Target value (real value), predicted value, the average of experimental values, and number of data points, respectively.

### 2.3 Adaptive neuro-fuzzy inference system (ANFIS)

Adaptive neuro fuzzy inference system is an integration between fuzzy inference system (FIS) and ANN. ANFIS is used to detect complex and non-linear relationships [60]. Fuzzy logic is a method of communicating ambiguous information. (FIS) employ membership functions, which are functions that utilize fuzzy logic to deduce information and connections between input and output variables [61]. However, the optimization and training of these membership functions is done through a manner similar to an ANN structure.

**Fig. 1:**
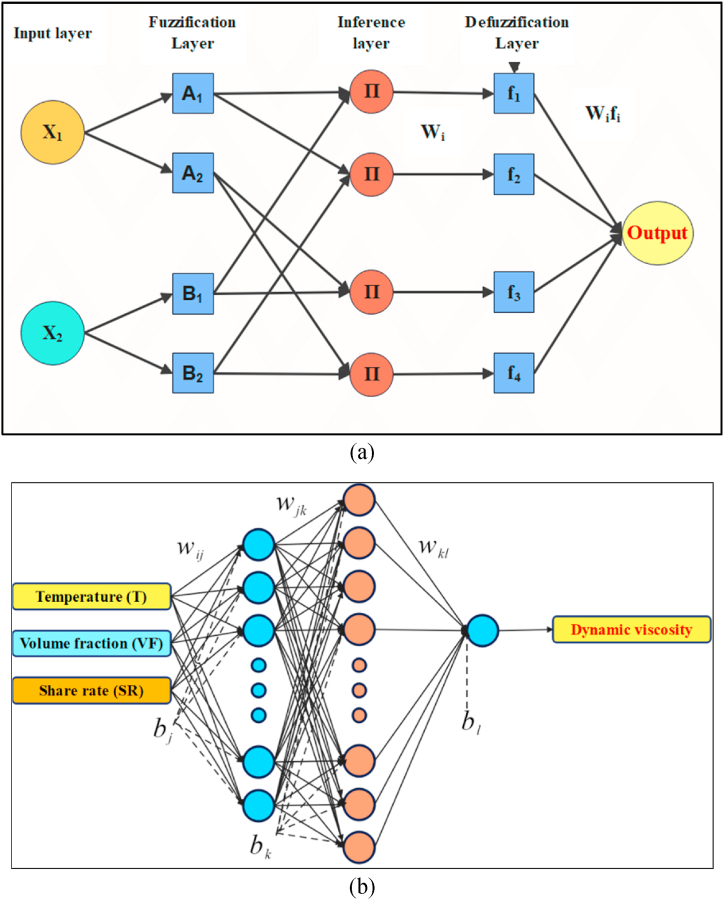
Basic general structure of a two input with two membership functions ANFIS model (a) and configuration of the proposed neural network (b).

Fig. 1(a) shows a basic structure of an ANFIS model with two inputs and two membership functions to illustrate the basic algorithm of ANFIS model. X_1_ and X_2_ are considered input variables which are fed to the first layer. The values are passed to the next layer through functions called membership functions. The input values are fuzzified and given a new value in the second layer (fuzzification layer). There are many types of membership functions. A well-known and used example in literature is called the gaussian function (Eqn. (11)).

(11) $$f\left(x\right)=e^{-\frac{1}{2}{\left(\frac{x-c}{\sigma }\right)}^{2}}$$

where, x is the value passed by the input layer, c is the center of the parameter fed to the function and σ is the variance parameter. In the third layer (inference layer) the model adjusts the values through a logical and operation which is described as in Eqn. (12) [60].

(12) $$W_{i}=A_{i}\left(x_{1,i}\right)B\left(x_{2,i}\right),i=1,2,..,n$$

The fourth layer is called defuzzification layer, where the fuzzified values are defuzzified and brought back to non-linguistic values. This is done through a product of output membership functions $\left(f_{i}\right)$ and the values of each node in previous layer (Eqn. (13)) [60].

(13) $$W_{i}f_{i}=W_{i}\left(m_{i}X_{1}+n_{i}X_{2}+r_{i}\right)$$

where, $m_{i}$, $n_{i}$ and $r_{i}$ are linear parameters that the ANFIS model optimizes with each iteration (epoch). Lastly, the value of each node is used to obtain a final result and prediction (Eqn. (14)) [60].

(14) $$output=\frac{\sum_{i}W_{i}f_{i}}{\sum_{i}W}$$

It is important to note here, some ANFIS models include an extra layer embedded between the fuzzification layer and inference layer which is the normalization layer. Which is done for same reasons of normalization mentioned in ANN.

## 3 SVR, ANN and ANFIS configurations

In this section, the detailed configurations of the SVR, ANN and ANFIS methods are introduced as applied in this study.

### 3.1 SVR configuration

In the present SVR model, the input parameters are the temperature, shear rate (SR) and volume fraction (VF) of the nano-fluid. The data is cross validated using the k-fold method. In this study, the dataset is divided into five equal-sized folds or subsets. The model is trained and evaluated five times, where in each iteration, four folds are used for training and one-fold is used for testing. This cross validation is performed to ensure the fitting of the model and ensures that the model found does not overfit the training data. Four types of non-linear kernel functions are studied. Then, the best performing function was further tuned to optimize its performance. The evaluation of the different kernel functions and their parameters is expressed in terms of the mean squared error (MSE) and the coefficient of determination (R^2^). The optimal parameters of the best kernel functions are discussed in Section 4.1.

### 3.2 ANN configuration

**Fig. 2:**
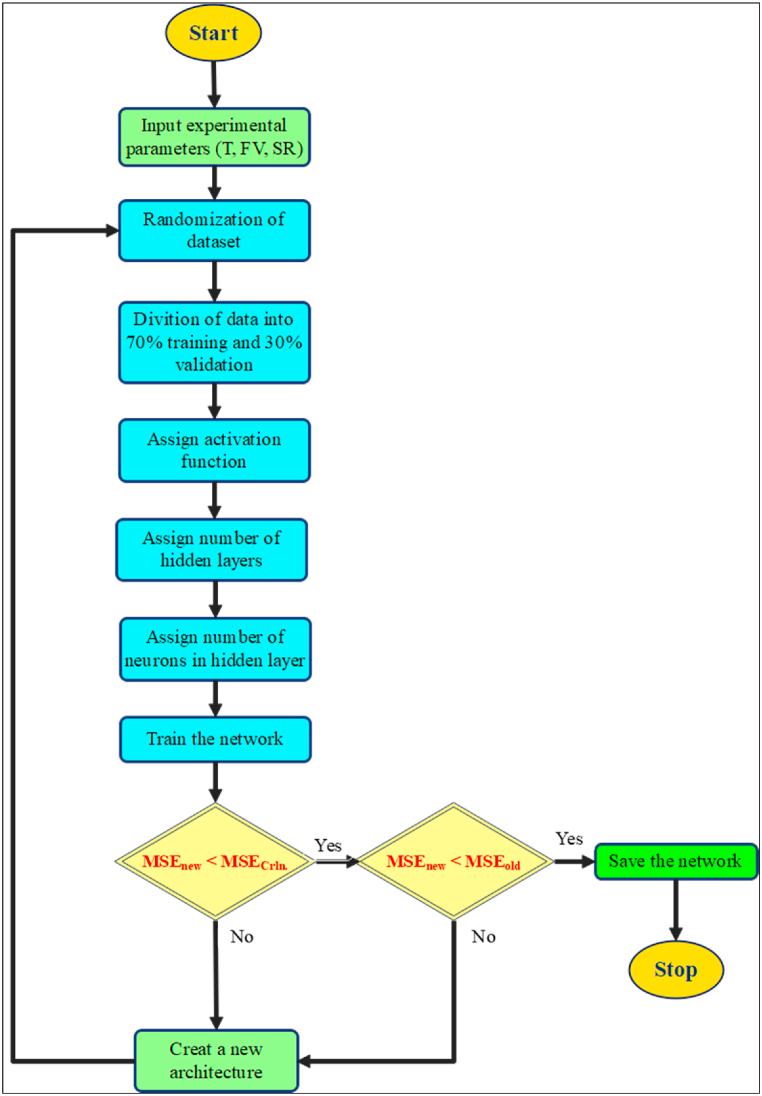
Procedures to obtain the optimized ANN model.

It is important to mention that machine learning and ANN models are only able to predict accurately within the range of input data fed to the model during training. Therefore, in this study, a total of 540 experimental data points available in Refs. [50,62] are used for the ANN model. A temperature range of −19.70 °C–70 °C is covered in these data at three different volume fractions (0%, 0.6%, 1%). Although the shear rate range is different for each volume fraction the total range of the shear rate starts from 6.42 1/s to 994.50 1/s. The data is divided into three groups: (1) training data (70%), (2) validation data (15%), and (3) testing data (15%). For the neural network structure, three input variables are used which are temperature, shear rate, and volume fraction of the nano-fluid as shown in Fig. 1(b). Then, various algorithms were examined at a single hidden layer with ten neurons to select the best training algorithm for the current ANN model. After that, different configurations were tested to optimize the ANN structure performance. The number of data points is proportional to the performance of the model. However, in some cases less learning datapoints may be better for the model depending on the nature of the data. Table 2 shows a list of studies where it can be noted that in the case of the ANN having same inputs and number of hidden layers the performance the ANN is improved with more data points used. However, in other cases where the number of input parameters are increased, it could achieve similar performance with less data. As the number of the available datapoints is relatively high (compared to that used in the literature, see Table 2), the optimal structure of the present study is a couple of hidden layers where in the first later 16 neurons are present and in the second layer 20 neurons are used. The detailed procedures that were performed to obtain the optimal ANN structure are depicted in Fig. 2. Further details about the ANN configuration and its evaluation will be discussed in Section 4.

**Table 2:** Configuration of the ANN used to predict the thermodynamic properties of nanofluid.

Refs.	# Input(s)	# Output(s)	# of data points	# of hidden layers	# of neurons	R^2^
[63]	3	1	128	2	[6,6]	0.9999
[64]	5	1	712	1	18	0.9930
[65]	7	1	715	2	[10,15]	0.99997
[66]	2	1	33	1	4	0.99687
[67]	4	1	185	2	[5,5]	0.95600
[40]	5	1	100	2	[5,10]	1.00000
[68]	2	1	28	2	[5,5]	0.99900
[69]	3	1	72	2	[5,2]	0.99850
[70]	3	2	15	1	4	0.98417

### 3.3 ANFIS configuration

The ANFIS model in this study, similar to the SVR and ANN models uses temperature, SR, and VF as input variables into the model to predict the dynamic viscosity of the nano fluid. The data is randomly divided into two main groups: 430 for training, and 107 for validation and testing (80% training and 20% validation and testing). Similar to ANN and SVR, this step protects the model from overfitting over the training data. Then the model is tested at four different membership functions at the same number of membership functions for each input (same membership functions configuration). These four membership functions are Gaussian, trapezoidal, triangular, and sigmoidal membership functions. These are the most common membership functions in literature which showed acceptable results. The FIS model is generated in many different methods. However, the method used in this study is grid partition which is essentially that the FIS model will generate rules based on the membership functions and divide the data into arbitrary partition. Finally different membership functions configurations are studied up to six membership functions for each input. The performance is evaluated using R^2^ and MSE. Results are shown and discussed in Section 4.3.

## 4 Results and discussions

The basic results of the SVR, ANN, and ANFIS based models are shown and discussed in Section 4.1, Section 4.2, and Section 4.3, respectively. Then, the performance of the ANN is compared with the correlation-based predictions in Section 4.4. Finally, the relationships between the dynamic viscosity parameters are discussed in Section 4.5.

### 4.1 SVR evaluation

As mentioned above, the SVR model depends heavily on the kernel function used to map the input data to output. Thus, four kernel functions, including two polynomial functions (quadratic and cubic) and two Gaussian functions (fine and medium), were examined to determine their effectiveness in accurately capturing the viscosity behavior. Table 3 shows the performance of the different kernel functions in terms of R^2^ and MSE on the validation set. It is shown that the polynomial cubic kernel function performs the best in both R^2^ and MSE. Thus, the cubic polynomial was chosen for the SVR model.

**Table 3:** Performance metrics of different kernel functions on the validation dataset.

Kernel function	R^2^_Validation_	MSE _Validation_
Polynomial (Quadratic)	0.96	0.0027256
Polynomial (Cubic)	0.97	0.0021800
Gaussian (fine)	0.96	0.0025036
Gaussian (medium)	0.91	0.0060604

Further tuning and optimization was implemented to the parameters of the cubic-based model to obtain better results. The highest R^2^ achieved was 0.98 and MSE of 0.0015979. Table 4 shows the optimum parameters found for the proposed support vector regression model.

**Table 4:** Optimal parameters and metrics for the proposed SVR model.

Model parameter	Optimal value
Kernel function	Polynomial (cubic)
Kernel Scale	0.22094
Bias	0.11801
K-folds	5
Epsilon (ε)	0.01257
Box constraint	0.12573
R^2^	0.98
MSE	0.0015979

**Fig. 3:**
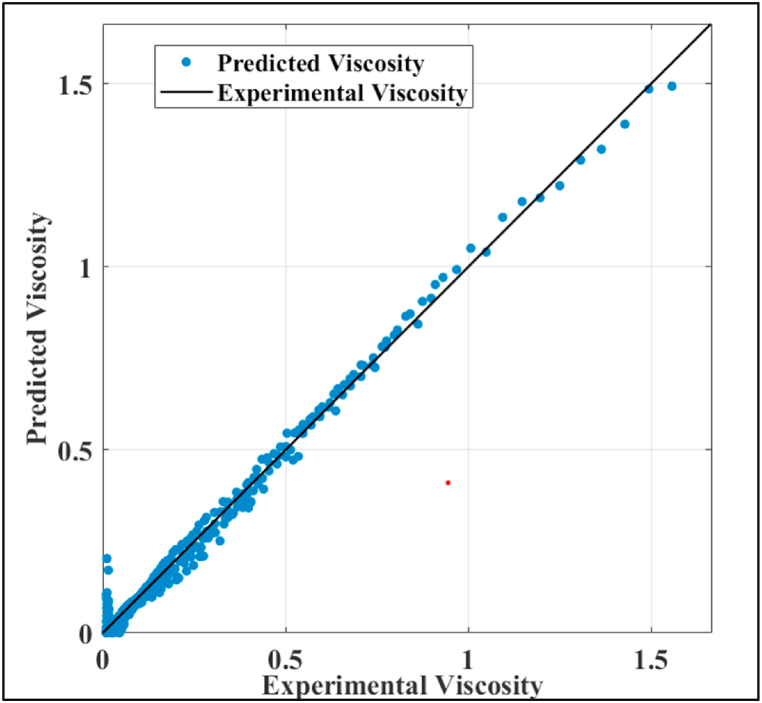
Regression diagram of cubic kernel function SVR model proposed.

Fig. 3 displays a graphical representation of the of the regression performance of the proposed SVR model. It is noted that the predicted and experimental values are in compliance with one another. However, an inferior performance is noted in the lower range of the viscosity (0–0.1) as the error is higher than at higher viscosity values.

**Fig. 4:**
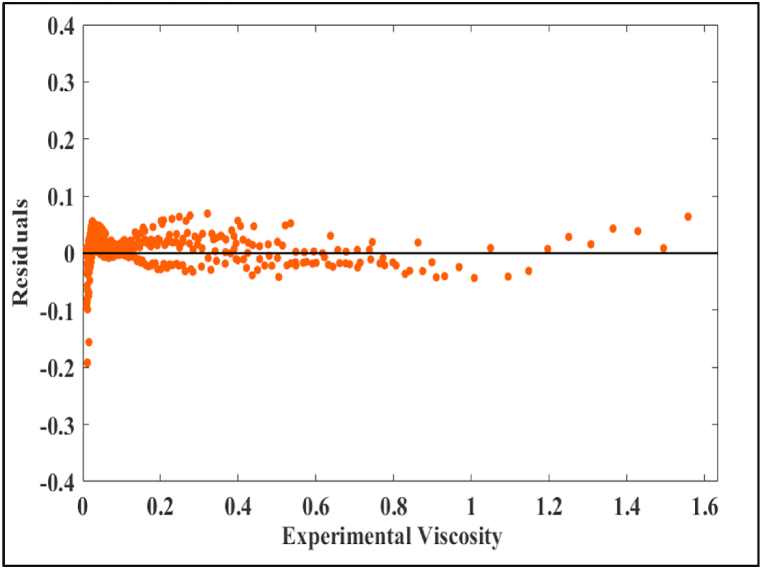
Residuals of the optimum SVR model (cubic).

Fig. 4 shows a representation of the residuals. The errors become more pronounced and reach undesirable levels when dealing with very low viscosity, indicating that the model's reliability diminishes at lower viscosity values.

### 4.2 ANN evaluation

There are several algorithms available in MATLAB that could be used to train the ANN configuration as shown in Table 5. It is quite challenging to predict which training algorithm will be faster and more accurate for a given problem. Several factors affect the convergence speed and accuracy of each algorithm. Factors such as the target problem, available training data, ANN size, convergence criteria, and task type (classification or regression) impact the convergence speed and accuracy of each algorithm. A straightforward approach to identify the best algorithm is to test all of them for a benchmark ANN configuration (e.g., 1 hidden layer, 10 neurons) as the prediction of the dynamic viscosity only needs three input parameters. Based on that, the MSE and R^2^ of these algorithms are listed in Table 5. A minimum error of MSE of 1.96 × 10^−6^ with R^2^ of 1.0 is noted for Levenberg-Marquardt algorithm. Thus, this algorithm is used for the training of the ANN in this study.

**Table 5:** Performance summary for training algorithms of the ANN model (single layer and ten neurons).

Algorithm	MSE	R^2^
Levenberg-Marquardt	1.96E-06	1.00000
Bayesian Regularization	2.40E-06	1.00000
BFGS Quasi-Newton	4.14E-04	0.99300
Resilient Backpropagation	7.25E-04	0.98771
Scaled Conjugate Gradient	7.60E-04	0.98787
Conjugate Gradient with Powell/Beale Restarts	4.31E-05	0.99927
Fletcher-Powell Conjugate Gradient	5.06E-04	0.99140
Polak-Ribiére Conjugate Gradient	6.50E-03	0.88285
One Step Secant	2.80E-03	0.96091
Variable Learning Rate Gradient Descent	8.09E-04	0.98661
Gradient Descent with Momentum	1.68E-02	0.65602
Gradient Descent	1.69E-02	0.65406

Once the best algorithm is selected, the effect of less teacher data is examined on an ANN with ten neurons, where half and quarter of the data only was taken into training the neural network. Results demonstrate that while the ANN model can accurately forecast the viscosity using less learning data, the performance of the ANN model is slightly improved when using all of the data as shown in Table 6.

**Table 6:** Performance comparison when using less learning data.

#Data used	R^2^	MSE
540	0.9999	6.5957E-06
270	0.9999	7.9855e-06
135	0.9998	1.1112E-05

The optimal size of the ANN is determined by examining several configurations. Table 7 shows samples of the examined configurations with their performance in terms of MSE and R^2^ associated with the training, validation, and testing data. A neural network with two hidden layers and [16,20] neurons was used for the prediction of the dynamic viscosity as it yields the minimum MSE with the highest R^2^.

**Table 7:** Samples of the ANN performance indicators throughout the development of its configuration.

# Hidden layers	Number of Neurons	MSE (Training)	MSE (Validation)	MSE (Testing)	R^2^ (Training)	R^2^ (Validation)	R^2^ (Testing)
1	14	9.24744e^−06^	8.59491e^−06^	7.73876e^−06^	0.99993	0.99995	0.99992
1	18	6.93778e^−06^	6.26718e^−06^	1.32700e^−05^	0.99995	0.99993	0.99990
1	20	7.32064e^−06^	8.31538e^−06^	5.99927e^−06^	0.99995	0.99992	0.99995
2	[16,20]	4.50306e^−06^	9.14446e^−06^	8.74922e^−06^	0.99996	0.99994	0.99994
2	[12,20]	9.53530e^−06^	7.27074e^−06^	5.02784e^−06^	0.99993	0.99993	0.99997

**Fig. 5:**
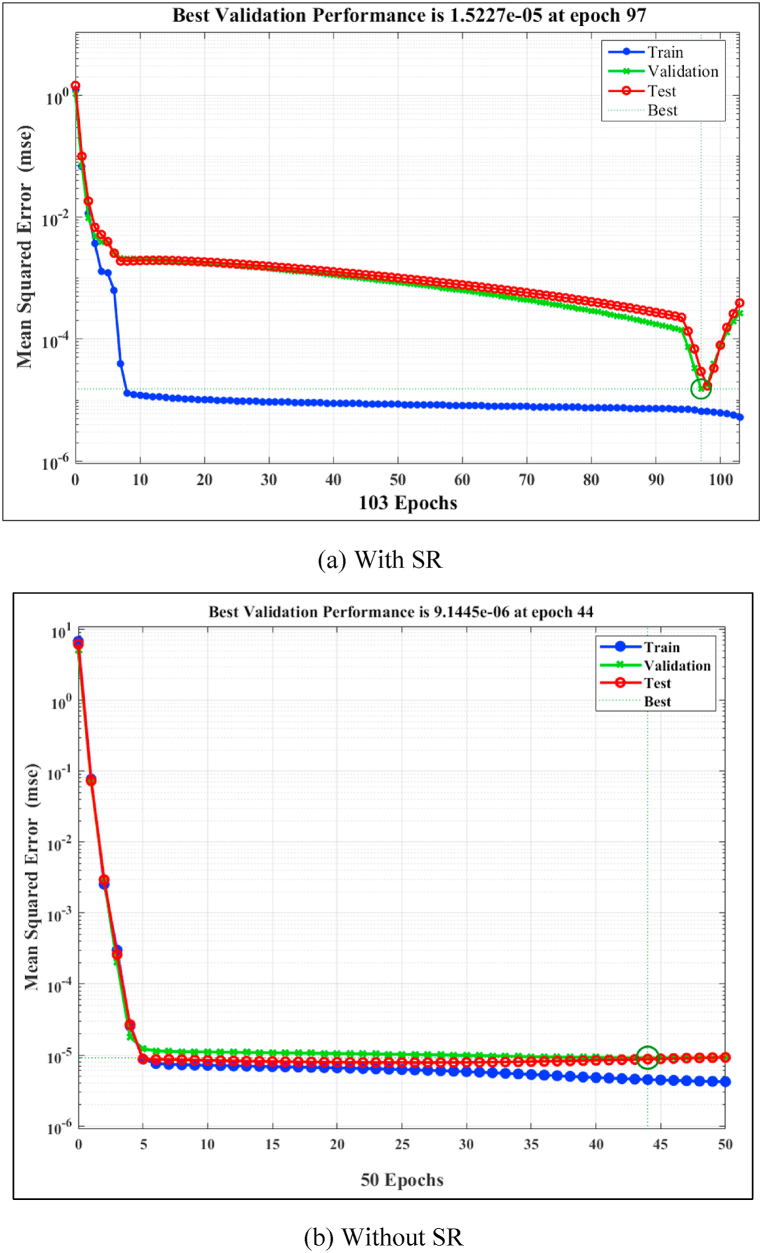
Network performance in terms of mean square error for the ANN (a) with SR, and (b) without SR as input variable.

The training process of the ANN alongside the validation and testing iterations (epochs) are depicted in Fig. 5. It is noticed that when SR is used as input parameter, the best validation performance is achieved at epoch 97 with MSE of 1.5227 × 10^−5^. After epoch 97, the MSE increases as shown in Fig. 5(a). Then the ANN is trained without SR, the best performance is achieved at epoch 44 with MSE of 9.1445 × 10^−6^ with slight increase in the MSE after epoch 44. In both cases, the MSE starts to decrease with more epochs until the MSE of validation data reaches a minimum value, then an overfitting is initiated with more epochs which increase the MSE. Therefore, the training process is terminated after six consecutive increases in the MSE (by default). It is also noted that the MSE of the validation data is closer to that of the training data for the case (Without SR, Fig. 5(b)) than for (With SR, Fig. 5(a)). Furthermore, the Mean Squared Error (MSE) is lower when SR is not used, suggesting that SR does not have a substantial impact on the dynamic viscosity and instead introduces noise to the Artificial Neural Network (ANN) model. Thus, it can be concluded that the behavior of the PAO-hBN nano-fluid is Newtonian (shear stress linearly proportion with SR). This explains the faster learning of the ANN when the SR is eliminated from the input layer.

**Fig. 6:**
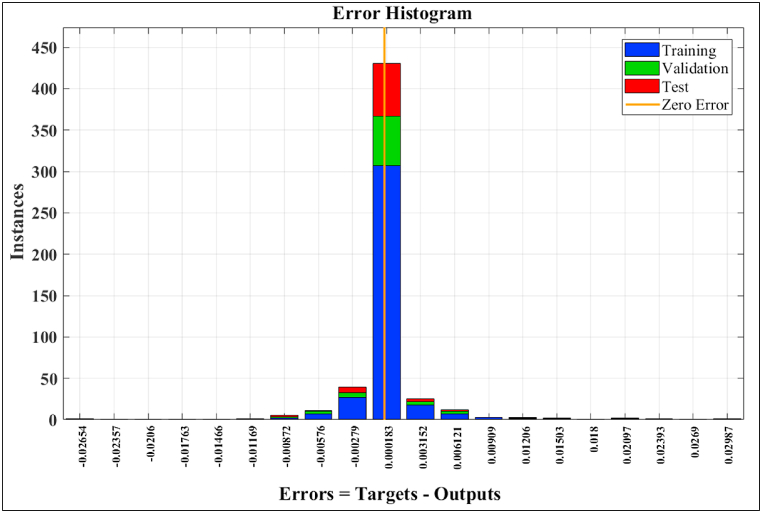
Error histogram for the proposed ANN model.

The error histogram is shown in Fig. 6. It represents the instances associated with error bins of the training (blue bars), validation (green bars), and testing data (red bars). It indicates that about 98% of errors fall between −0.00872 and 0.00909 while some of errors are out of this range.

**Fig. 7:**
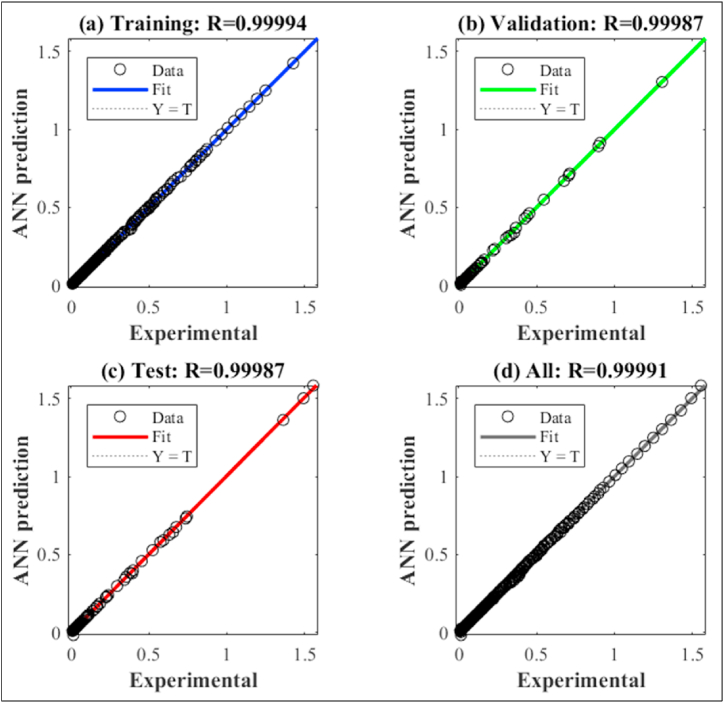
Regression diagram for the (a) training dataset, (b) validation dataset, (c) testing dataset, (d) all data set used.

Fig. 7 shows a graphical presentation of the regression performance for the ANN training, validation, and testing data. It is noticed that the R^2^ of the validation data is 0.0007 lower than the training data. This emphasis the superior performance of the selected ANN configuration which confirm its applicability and reliability to predict the dynamic viscosity of the PAO-hBN nano-fluids.

### 4.3 ANFIS model evaluation

As mentioned in Section 2.3, the type of membership functions used in the ANFIS model is an essential part of the model development. Table 8 shows the performance of four different membership functions on same configuration (3 MFs for each input). It is worth noting that all evaluated types of membership functions demonstrate acceptable MSE and R^2^ values, with the exception of the trapezoidal membership function. This is attributed to its limited ability to accurately capture the non-linear behavior exhibited by the various input parameters. Furthermore, the triangular membership function shows the best performance, outperforming Gaussian slightly. Thus, it has been chosen as the membership function for the input variables of the model.

**Table 8:** Performance comparison for different membership function types with three membership functions per input.

MFs	RMSE training	MSE training	RMSE validation	MSE validation	R^2^
Triangular	0.002278	0.51892e^−05^	0.00585804	3.43166E-05	0.9998
Gaussian	0.003622	1.31189e^−05^	0.02350200	0.000552344	0.9981
Sigmoid	0.004121	1.69826e^−05^	0.01011640	0.000102342	0.9995
Trapezoidal	0.090390	.00817035	0.10124000	0.010249538	0.8673

After the selection of the best membership function an optimal configuration must be selected to obtain the optimal performance for the ANFIS model. Table 9 shows the performance measures for the different configurations tested. It is discussed that as the number of membership functions increases the performance of the ANFIS model is improved. However, the computational procedures become more complex and take more computing and training time. Furthermore, as the number of membership functions increases, the more that model overfits the training data. As shown in Table 9, although the MSE for training for 6 membership functions is lower than that of 5, the MSE over the validation data for 5 membership functions is lower than using 6 membership functions. This can also be shown through the R^2^ value decrease between 5 and 6 membership functions for each input. Thus, five membership functions for each input have been selected as the optimum number of membership functions for the model. These membership functions are shown in Fiig. 8(a) SR membership functions, (b) temperature membership functions (c) VF membership functions (See Supplementary Material).

**Table 9:** Performance of different configurations for input membership functions.

MFs	RMSE training	MSE training	RMSE validation	MSE validation	R^2^
3	0.002278	5.18928e^−06^	0.00585804	34.3166e^−06^	0.9998
4	0.001934	3.74036e-^06^	0.00213885	4.57468e^−06^	0.9999
5	0.000782	0.611524e^−06^	0.00123230	1.51856e^−06^	1
6	0.000389	0.151321e^−06^	0.00477786	22.8279e^−06^	0.9999

Fiigure 9 (See Supplementary Material) illustrates the proposed model’s performance and its agreement with experimental values across range of the data. It is noted that the performance of the ANFIS model is consistent and is able to predict accurately over the full range of the data. Finally, the ANFIS model shows a similar performance to the ANN model developed. However, the computation and training time required in the ANFIS model is much higher and demands more computational capabilities than that of the ANN. Therefore, the ANN can be recommended as an accurate and practical choice for prediction of dynamic viscosities of the PAO-hBN nanofluid.

### 4.4 Comparative evaluation of ANN model vs. correlation

Sleiti [50] has developed an empirical correlation, represented by Eq. (15), to predict the dynamic viscosity (μ_nf_) of PAO-hBN nanofluids. In the current study, this correlation serves as a benchmark for evaluating the accuracy of the machine-learning methods developed.

(15) $$\mu_{nf}=Ae^{\left(BT+C\emptyset^{2}\right)}$$

where *A*, *B*, and *C* are characteristic parameters of the nano-fluid which are calculated based on the temperature range of the fluid. Four temperature ranges were considered (253–273 K, 273–293 K, 293–313 K, and 313–383 K) with specific values for each range were assigned to the parameters (*A*, *B*, and *C*). This temperature-based clustering was performed to improve the performance of the correlation as a single value for each parameter cannot fit with all data over the full temperature range. However, in the ANN model, the same configuration is sufficient to predict the viscosity regardless of the temperature range. Furthermore, the accuracy of prediction using the ANN is superior compared to the correlation as shown in Fiig. 10 (See Supplementary Material). It is noticed that the ANN yields good agreement with the experimental data at any temperature or volume fraction (VF) value. However, the correlation gives random deviation from the experimental data with the variation of the VF, which is mostly noticeable at VF of 0.60% (see Fiig. 10(b)) (See Supplementary Material). Also, the correlation-based model gives higher absolute relative error (ARE), which exceeds 11% for some points as shown in Fiig. 10(a) (See Supplementary Material). However the correlation-based model seems to outperform the ANN at higher values of VF (1%) as shown in Fiig. 10(c).

In general, the ANN predicts the dynamic viscosity with lower error than of the correlation over a significant part of the temperature range. From Fiig. 10 (See Supplementary Material), it is clear that the performance of the ANN is superior to the correlation up to temperature of 45 °C. At higher temperatures than 56 °C, the performance of the ANN is poor compared to that of the correlation model. Thus, further improvements for the ANN are needed such as (i) training the ANN with prior manual clustering of the experimental data, or/and (ii) eliminating the SR from the input layer.

### 4.5 Relationship between the parameters of the nano-fluid

The ANN is modified by eliminating the SR from the input layer and training the model with the same structure [two hidden layers (16,20)].

As shown in Fiig. 11(b) (See Supplementary Material), the new ANN model is more accurate than the correlation over the full temperature range. This addresses the deficient performance of the previous ANN model at temperatures higher than 45 °C (Fiig. 11 (a)) (See Supplementary Material). Furthermore, the performance of the ANN is improved compared to the 3-input-based ANN model over the full temperature range with absolute relative error less than 1.89% (Fiig. 11 (b)) (See Supplementary Material).

The enhanced performance of the ANN prediction model upon removing SR suggests that the behavior of PAO-hBN nano-fluids can be considered Newtonian in nature. That is, the shear stress linearly proportional with shear rate with constant slope (viscosity) in ideal case. This implies that temperature and concentration are parameters affecting the dynamic viscosity of the PAO-hBN nano-fluid (Fiig. 12(a)) (See Supplementary Material) and slightly changes with shear rate (Fiig. 12(b)) (See Supplementary Material), which agrees with the experimental findings of [50]. However, it should be noted that the model's training data only consists of information about the fluid's behavior when it is Newtonian. Therefore, the model may not accurately predict the viscosity of non-Newtonian fluids or when the fluid exhibits non-Newtonian behavior. To improve the model's accuracy in such scenarios, additional training data from a wider range of fluid behaviors may be necessary. Additionally, incorporating other relevant physical properties of the fluid into the model may also improve its predictive capability. Therefore, it can be recommended to eliminate the SR from the input layer of ANN models for Newtonian fluid to enhance its accuracy.

## 5 Conclusions

In this study, three machine learning-based methods were examined to predict the dynamic viscosity of Polyalpha-Olefin-hexagonal boron nitride (PAO-hBN) nanofluids. These methods include support vector regression (SVR), artificial neural network (ANN) and adaptive neuro-fuzzy inference system (ANFIS). A total of 540 experimental data points is used for the training, validating, and testing the different methods. All three methods show acceptable prediction results. However, Both the ANN and ANFIS models outperformed (R^2^ > 0.99) the SVR model (R^2^ value did not exceed 0.98). Furthermore, unlike the ANFIS and ANN models, the SVR failed to conserve its accuracy across the full range of viscosity especially at lower values of viscosity where the SVR model loses its accuracy. An optimum ANFIS model was found in this study which includes three inputs, each input with five membership functions. The optimal membership function was found to be gaussian and triangular, where the triangular outperforms the Gaussian slightly. Although the ANFIS model gives equivalent results to the ANN (R^2^ > 0.99 and MSE <1 × 10^−6^), The ANN model was selected as a better model to predict the dynamic viscosity. Due to its robustness and responsiveness to training and learning, unlike the ANFIS model which took more training and learning time to achieve similar performance. The ANN model was trained over several training algorithms (available in MATLAB) and configurations were evaluated, then the Levenberg-Marquardt training algorithm was selected. The ANN model developed is shown to be more accurate than the correlation-based model up to temperature of 45 °C if the shear rate (SR) is taken as input parameter. However, elimination of the SR from the input layer improves the accuracy of the ANN model over the full temperature range (−19.7 °C–70 °C). Without SR, the absolute relative error (ARE) of the ANN model does not exceed 1.89% compared to 11% in the correlation-based model. Finally, it is recommended to eliminate the SR from the input parameters of ANN model for Newtonian fluids as it reduces the accuracy of the predicting process.

## 6 Future work

Future research in this area could involve the exploration of forecasting PAO-hbN nanofluids’ dynamic viscosity values using alternative models of machine learning. Decision tree-based models and deep learning models such as convolutional neural networks (CNNs) and recurrent neural networks (RNNs) are potential candidates for this purpose. Moreover, the predictive capabilities of the developed model could be extended to other properties of nanofluids such as thermal conductivity, specific heat, and density. This can be achieved by incorporating relevant input parameters such as particle size, concentration, and temperature into the model. Further experimental work could be conducted to validate the accuracy of the developed model and evaluate the effect of different parameters on the dynamic viscosity and other properties of PAO-hBN nanofluids. This would enable a deeper understanding of the behavior of these nanofluids under different conditions. Overall, the results of this study have demonstrated the potential of machine learning-based models for evaluating PAO-hBN nanofluids’ dynamic viscosity. The future research directions outlined here would expand the scope of this work and provide a more comprehensive understanding of the underlying physical mechanisms driving the behavior of nanofluids. This knowledge could lead to the development of more accurate and efficient models for predicting the properties of nanofluids, which could have significant implications for a wide range of industrial applications. Therefore, it is recommended that researchers in this field continue to explore and refine machine learning models for predicting the properties of nanofluids, while also conducting further experimental investigations to validate and extend these models.

## Author contribution statement

Yazeed AbuShanab: Ahmad K. Sleiti: Conceived and designed the experiments; Performed the experiments; Analyzed and interpreted the data; Contributed reagents, materials, analysis tools or data; Wrote the paper.

Wahib A. Al-Ammari: Analyzed and interpreted the data; Wrote the paper.

Samer Gowid: Analyzed and interpreted the data; Contributed reagents, materials, analysis tools or data; Wrote the paper.

## Data availability statement

Data will be made available on request.

## Declaration of competing interest

The authors declare that they have no known competing financial interests or personal relationships that could have appeared to influence the work reported in this paper.

## Nomenclature

### Abbreviation

AI: Artificial Intelligence
ANFIS: Adaptive Neuro-Fuzzy Inference System
ANN: Artificial Neural Network
BR: Bayesian Regularization
BRT: Boosted Regression Tree
EGB: Ethylene Glycol-Based
FIS: Fuzzy Inference System
LM: Levenberg-Marquardt
MFs: Membership Functions
MWCNT: Multi-Walled Carbon Nanotubes
OB: Oil Based
PAO-hBN: Polyalpha-Olefin-hexagonal Boron nitride Nanofluids
PGDNN: Physics-Guided Deep Neural Network
RP: Resilient Propagation
RSM: Response Surface Methodology
SVR: Support Vector Regression
SVM: Support Vector Machine
VF: Volume Fraction
WB: Water-Based

### Symbols

$\mu_{nf}$: Dynamic viscosity of the nano-fluid
T: Temperature of the nano-fluid
∅: Volume fraction of the nano-fluid
w: Weight coefficient
b: Bias
ε: Error
ξ: Slack variable
$m_{i}$, $n_{i}$: Linear parameters for the ANFIS model
R^2^: Coefficient of determination
ARE: Absolute Relative Error
MSE: Mean Squared Error
