# Supplementary material for: Accurate prediction of dynamic viscosity of polyalpha-olefin boron nitride nanofluids using machine learning
Source: Heliyon. 2023 May 26;9(6):e16716. doi: 10.1016/j.heliyon.2023.e16716 (PMC10245067; doi:10.1016/j.heliyon.2023.e16716)
Supplement: Multimedia component 1 [file mmc1.docx]

**Supplementary Material**

**Accurate prediction of dynamic viscosity of Polyalpha-Olefin nanofluids using machine learning techniques**

Yazeed AbuShanab^a^, Wahib A. Al-Ammari^a^, Samer Gowid^a^, Ahmad K. Sleiti^a*^

^a^ Department of Mechanical & Industrial Engineering, College of Engineering, Qatar University, Qatar

^*^Corresponding author: [asleiti@qu.edu.qa](mailto:asleiti@qu.edu.qa)


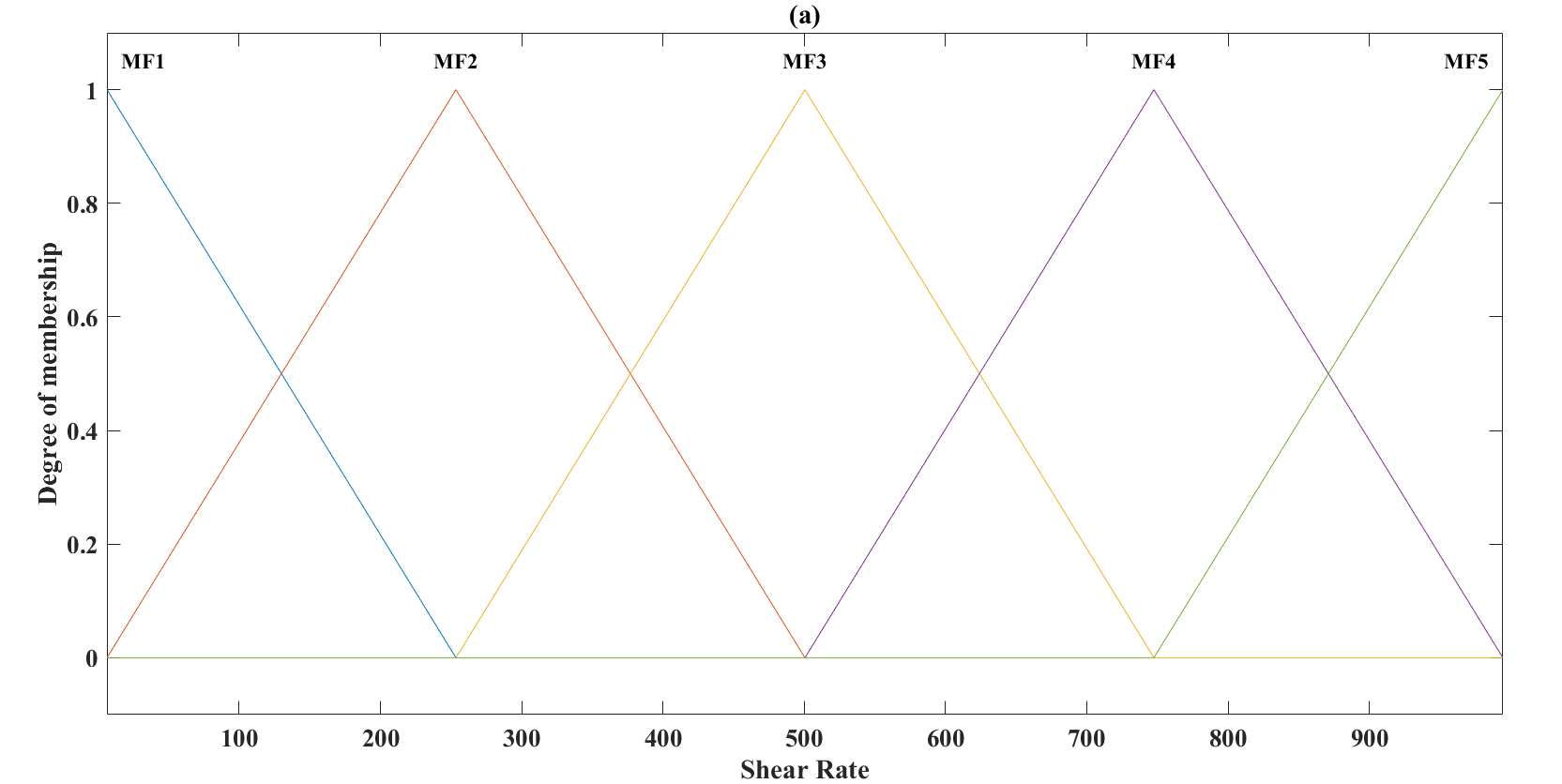


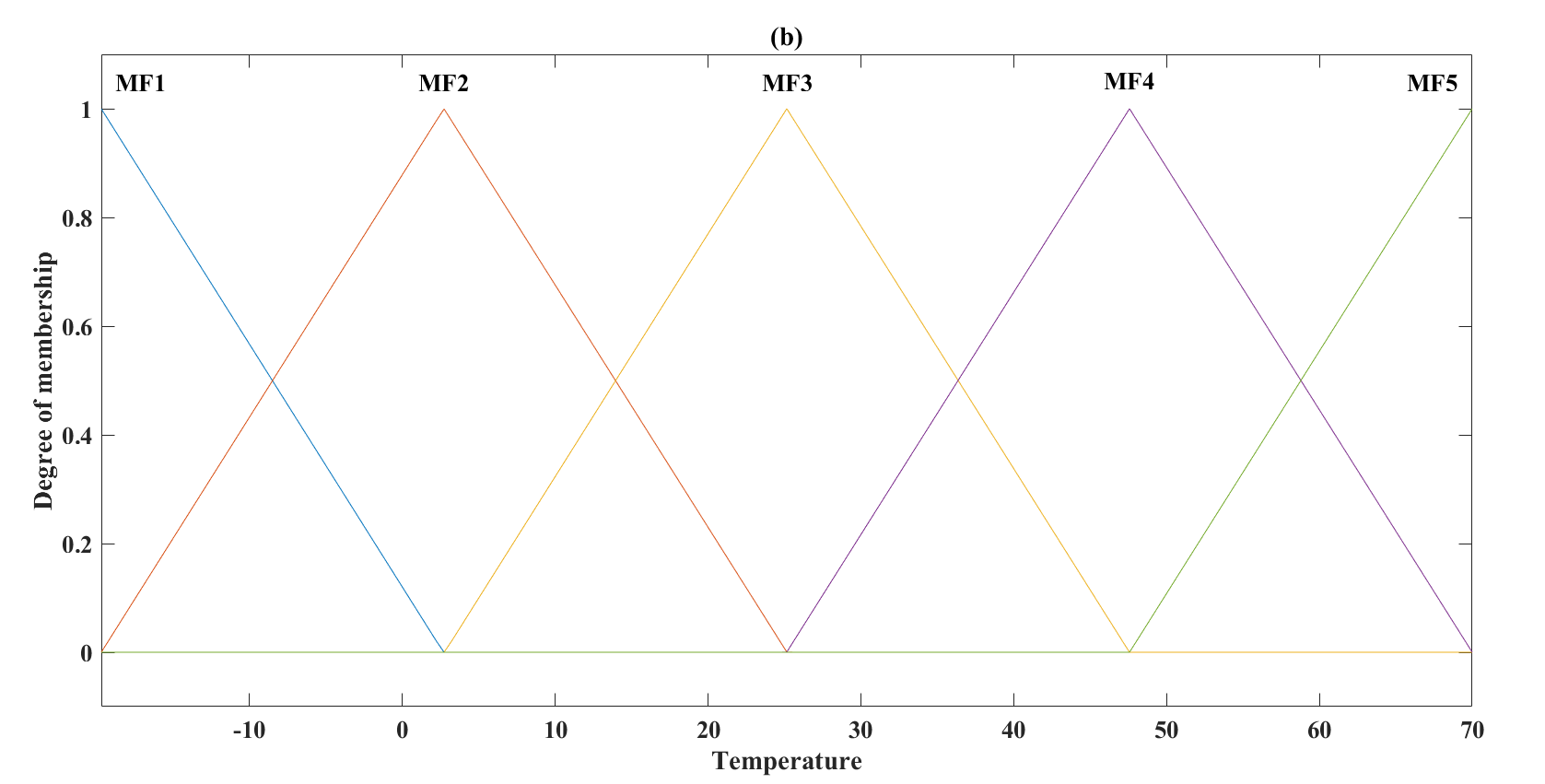


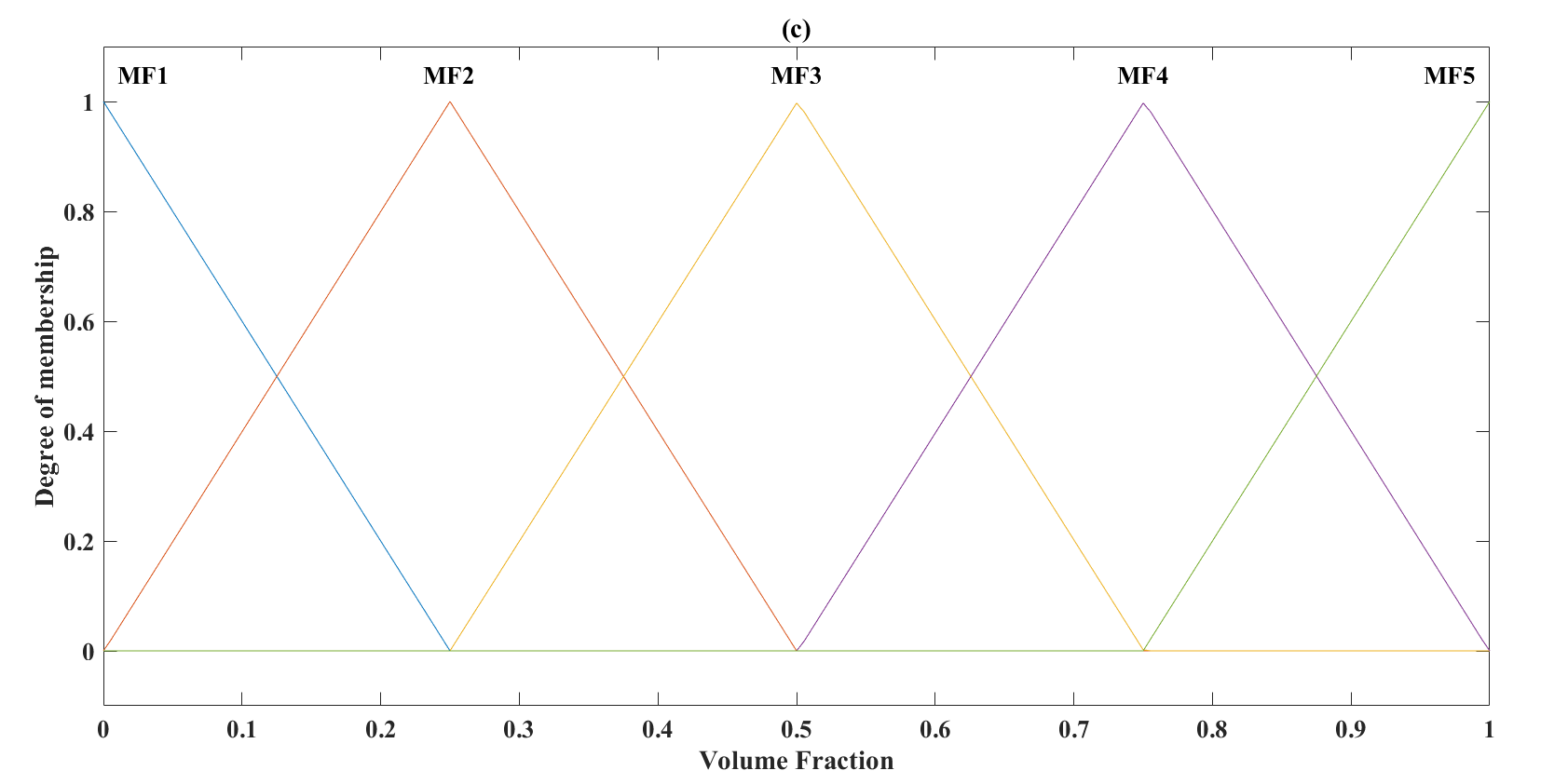


**Fig. 8**: Membership functions of the 3 inputs for the proposed ANFIS model; (a)shear rate, (b) temperature, and (c) Volume fraction


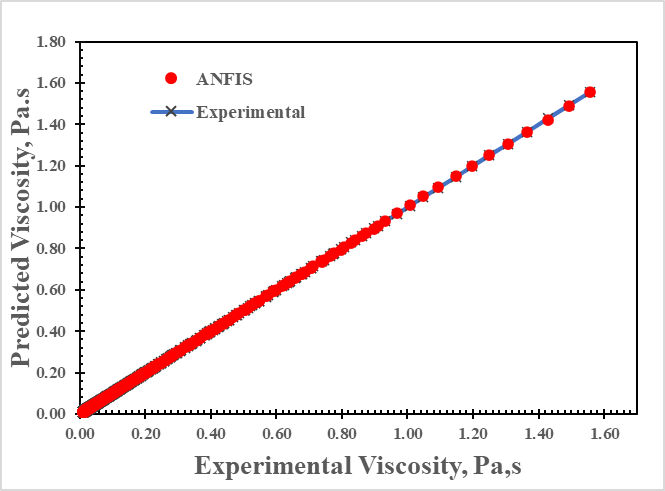


**Fig. 9**: Regression diagram of proposed ANFIS model over the whole Data range


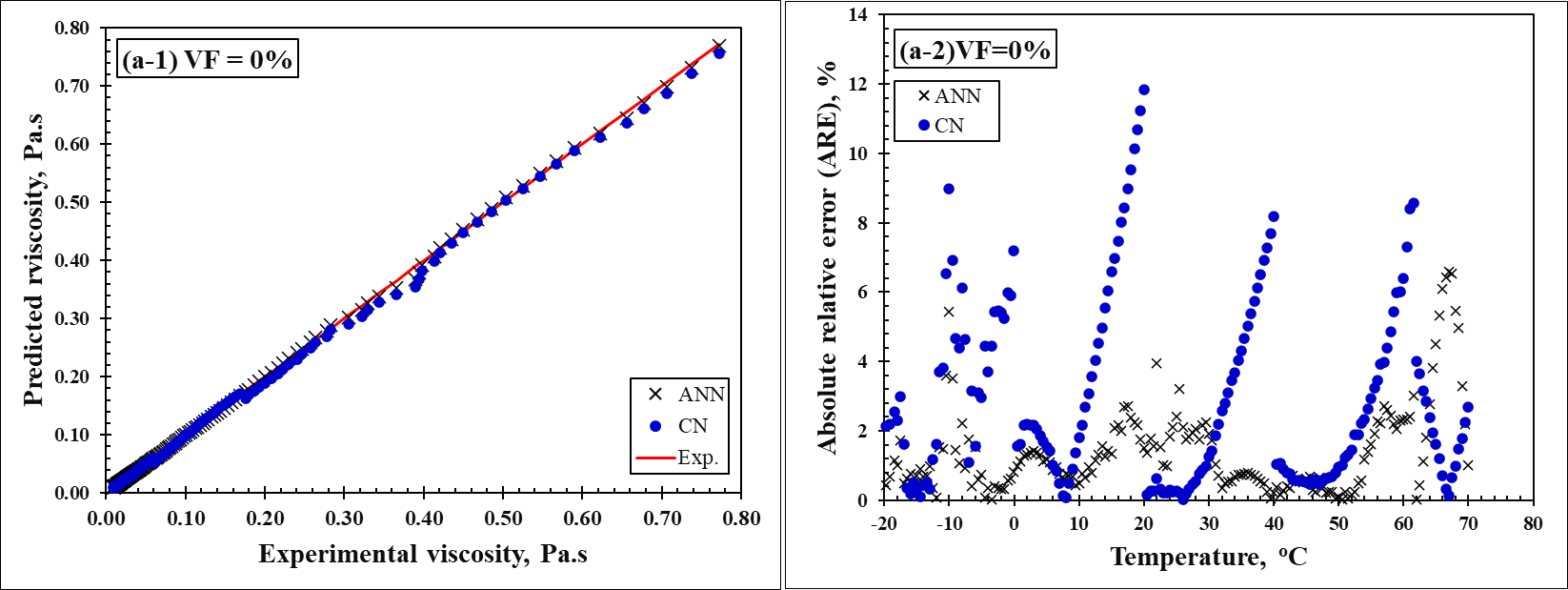


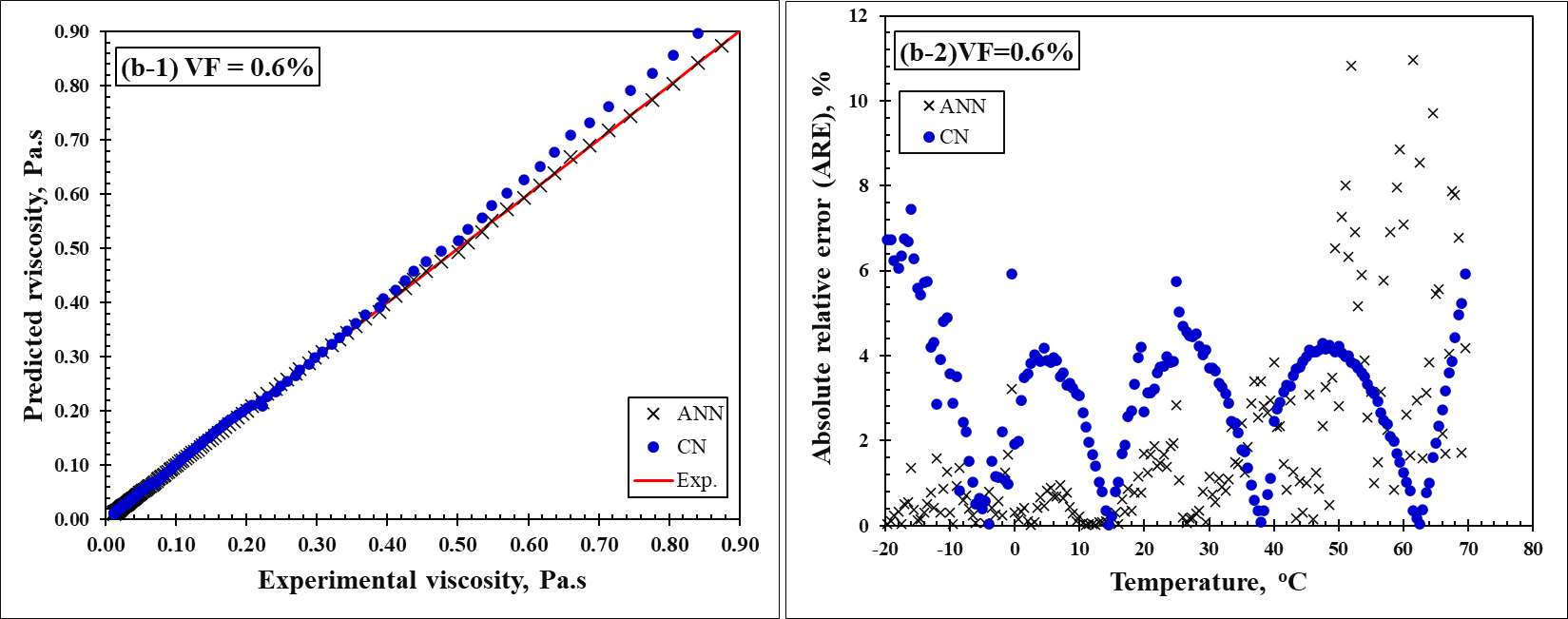


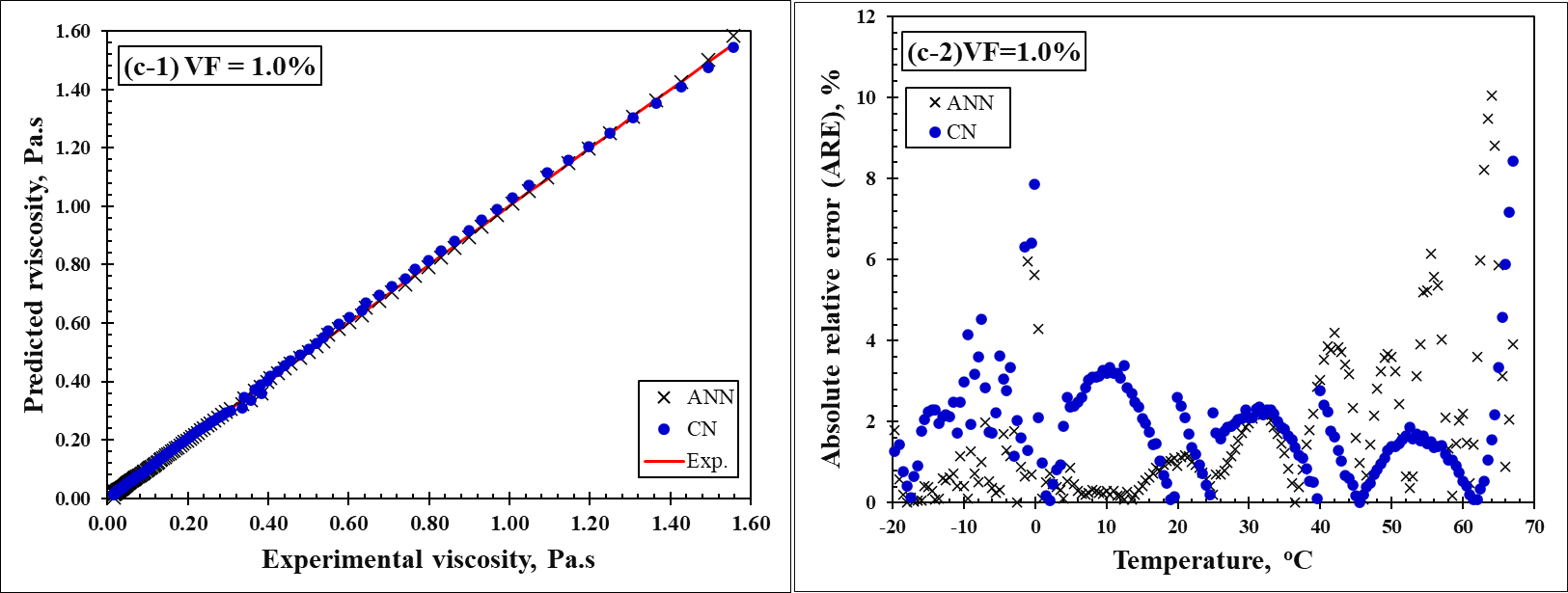


**Fig. 10.** Comparison between ANN model and correlation model (CN) at volume fraction of (a) 0.0%, (b) 0.60%, and (c) 1.0% using temperature, volume fraction, and shear rate as inputs for the ANN model.


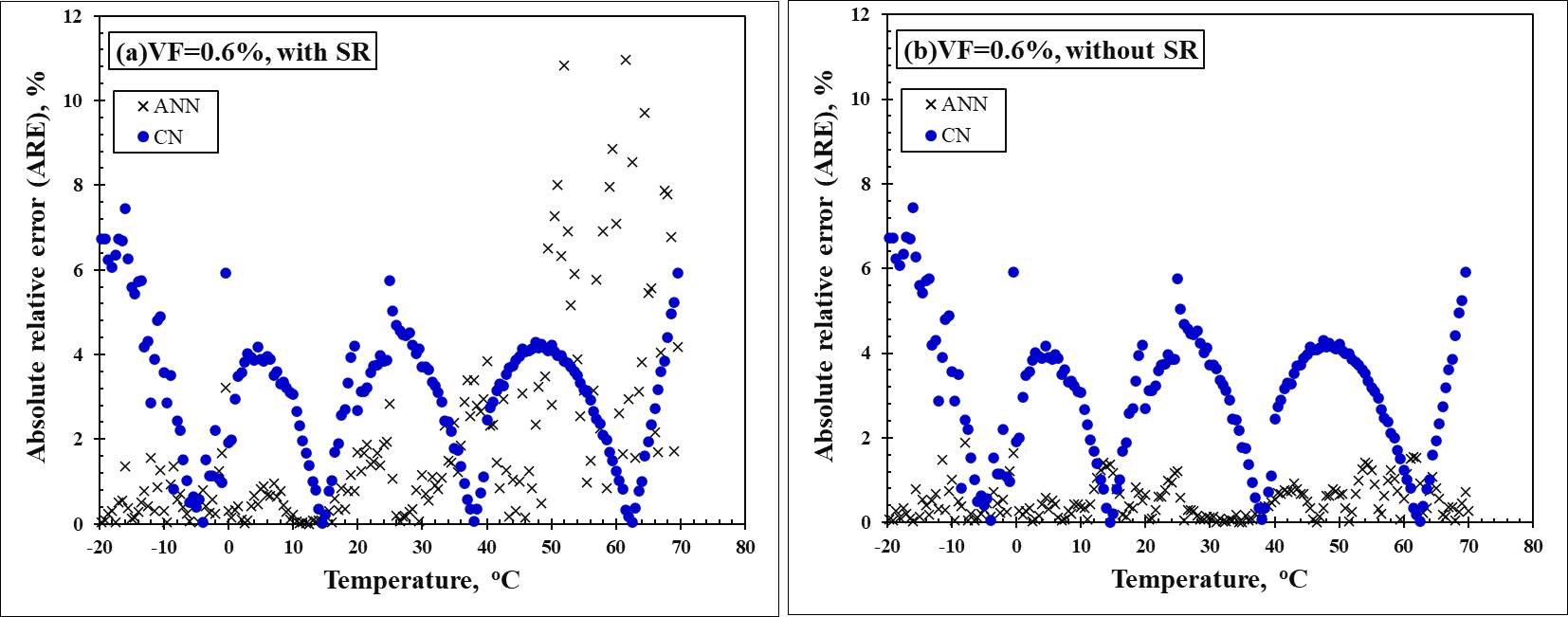


**Fig. 11.** Effect of using SR in the ANN configuration in terms of the absolute relative error. (a) ARE with SR, and (b) ARE without SR


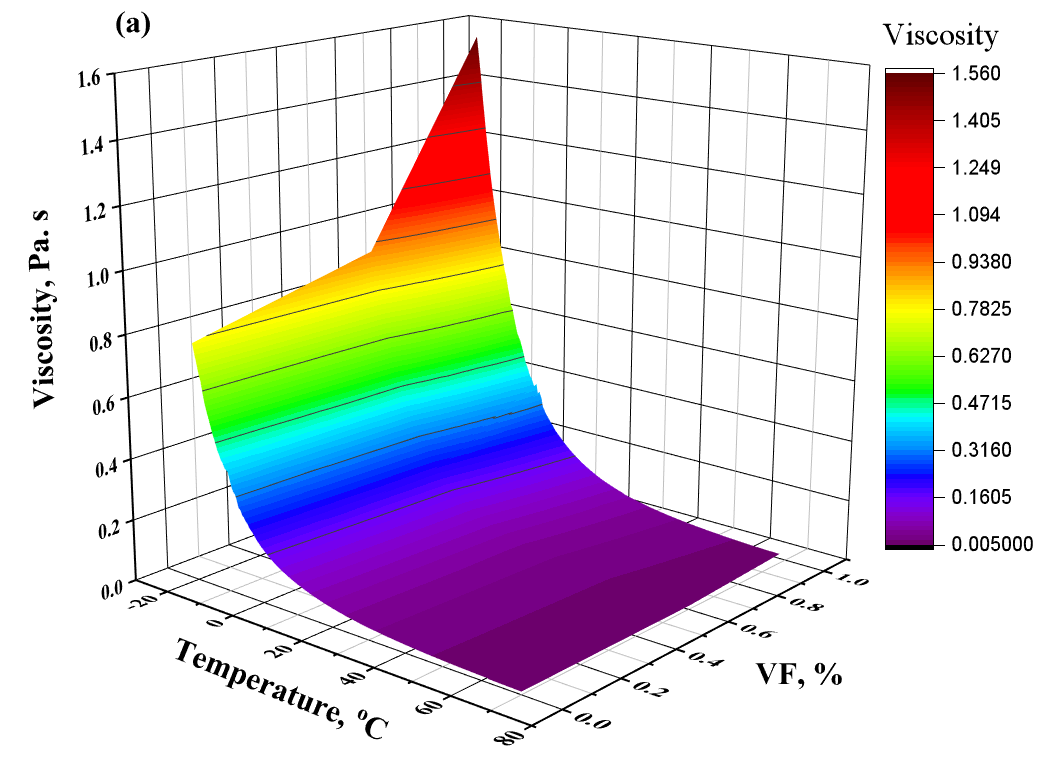


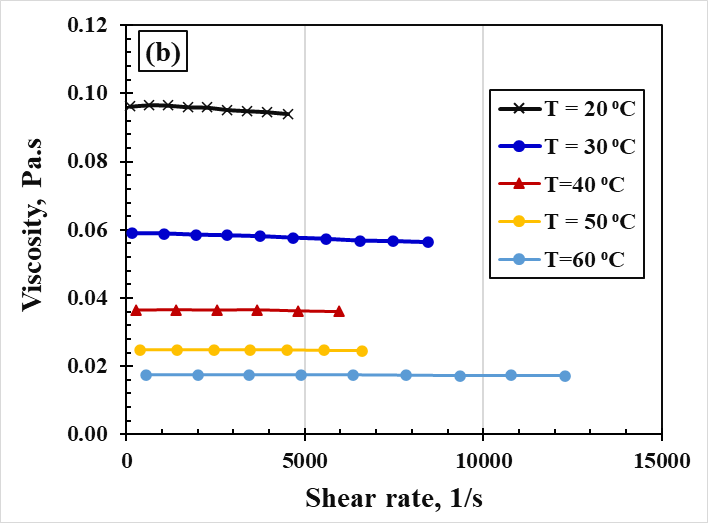


**Fig. 12.** The variation of the dynamic viscosity of the Polyalpha-Olefin- boron nitride nanofluid with the (a) temperature, and (b) shear rate at VF=1.0% and different temperatures.
